# Behavioral Outcomes and Neural Network Modeling of a Novel, Putative, Recategorization Sound Therapy

**DOI:** 10.3390/brainsci11050554

**Published:** 2021-04-27

**Authors:** Mithila Durai, Zohreh Doborjeh, Philip J. Sanders, Dunja Vajsakovic, Anne Wendt, Grant D. Searchfield

**Affiliations:** 1 Section of Audiology, School of Population Health, The University of Auckland, Auckland 1023, New Zealand; mithu.durai@gmail.com (M.D.); zohreh.doborjeh@auckland.ac.nz (Z.D.); philip.sanders@auckland.ac.nz (P.J.S.); d.vajsakovic@auckland.ac.nz (D.V.); 2 Eisdell Moore Centre, Auckland 1023, New Zealand; 3 Centre for Brain Research, The University of Auckland, Auckland 1023, New Zealand; 4 Knowledge Engineering & Discovery Research Institute, Auckland University of Technology, Auckland 1010, New Zealand; anne.wendt@aut.ac.nz; 5 Brain Research New Zealand—Rangahau Roro Aotearoa, The University of Auckland, Auckland 1142, New Zealand

**Keywords:** tinnitus perception, recategorizing, tinnitus avatar, psychoacoustic, environmental sounds, morphed sound, brain-inspired spiking neural network

## Abstract

The mechanisms underlying sound’s effect on tinnitus perception are unclear. Tinnitus activity appears to conflict with perceptual expectations of “real” sound, resulting in it being a salient signal. Attention diverted towards tinnitus during the later stages of object processing potentially disrupts high-order auditory streaming, and its uncertain nature results in negative psychological responses. This study investigated the benefits and neurophysiological basis of passive perceptual training and informational counseling to recategorize phantom perception as a more real auditory object. Specifically, it examined underlying psychoacoustic correlates of tinnitus and the neural activities associated with tinnitus auditory streaming and how malleable these are to change with targeted intervention. Eighteen participants (8 females, 10 males, mean age = 61.6 years) completed the study. The study consisted of 2 parts: (1) An acute exposure over 30 min to a sound that matched the person’s tinnitus (Tinnitus Avatar) that was cross-faded to a selected nature sound (Cicadas, Fan, Water Sound/Rain, Birds, Water and Bird). (2) A chronic exposure for 3 months to the same “morphed” sound. A brain-inspired spiking neural network (SNN) architecture was used to model and compare differences between electroencephalography (EEG) patterns recorded prior to morphing sound presentation, during, after (3-month), and post-follow-up. Results showed that the tinnitus avatar generated was a good match to an individual’s tinnitus as rated on likeness scales and was not rated as unpleasant. The five environmental sounds selected for this study were also rated as being appropriate matches to individuals’ tinnitus and largely pleasant to listen to. There was a significant reduction in the Tinnitus Functional Index score and subscales of intrusiveness of the tinnitus signal and ability to concentrate with the tinnitus trial end compared to baseline. There was a significant decrease in how strong the tinnitus signal was rated as well as ratings of how easy it was to ignore the tinnitus signal on severity rating scales. Qualitative analysis found that the environmental sound interacted with the tinnitus in a positive way, but participants did not experience change in severity, however, characteristics of tinnitus, including pitch and uniformity of sound, were reported to change. The results indicate the feasibility of the computational SNN method and preliminary evidence that the sound exposure may change activation of neural tinnitus networks and greater bilateral hemispheric involvement as the sound morphs over time into natural environmental sound; particularly relating to attention and discriminatory judgments (dorsal attention network, precentral gyrus, ventral anterior network). This is the first study that attempts to recategorize tinnitus using passive auditory training to a sound that morphs from resembling the person’s tinnitus to a natural sound. These findings will be used to design future-controlled trials to elucidate whether the approach used differs in effect and mechanism from conventional Broadband Noise (BBN) sound therapy.

## 1. Introduction

Tinnitus (“phantom sound”) is a highly prevalent condition affecting 6% of the New Zealand general population and 13.5% of over 65′s [1]. Tinnitus can have a catastrophic effect on 10–15% of those who experience it, manifesting as difficulties with sleep, concentration, anxiety/depression, hearing difficulties, and social association; and in extreme cases, has been associated with suicide [2].

The precise mechanisms giving rise to tinnitus perception and distress are still under study, although it is now generally understood to be a result of central compensatory processes following peripheral auditory deafferentation [3,4,5,6]. Increasing evidence suggests that final tinnitus magnitude judgments may involve complex interactions between detection of the signal, presence of external sound as well as significant influences from internal factors such as attention, memory, and emotion [7]. At a perceptual level, one hypothesis is that tinnitus is initially processed as an external sound within the auditory system, i.e., identical initial feature extraction and streaming as an auditory object [7,8]. However, as tinnitus characteristics do not completely match those of external sounds, attention may become diverted to it during the latter semantic stages of auditory object processing [7,8,9,10].

Constant attention processes diverted towards processing prediction errors may keep the tinnitus salient and possibly recruit tinnitus distress networks, eliciting negative reactions. The Adaptation Level Theory model of psychoacoustics posits the sensation that is attended to at any point in time will be given greater weighting and salience in perception [7,11]. Preliminary electrophysiological findings have observed cortical-level frequency-based auditory stream segregation may be disrupted among individuals with tinnitus at or near the frequencies corresponding to tinnitus pitch, observed as enhanced resting-state N1c and abnormal growth of N1c waveforms [12]. There were different levels of activity between tinnitus and control groups in regions corresponding to attentional networks (precentral gyrus, right superior temporal, insula, middle, and inferior frontal gyrus) as well as limbic regions (parahippocampus and insula). Disruption may occur within the ambiguous streaming region, in which attention or contextual alterations can bias perception towards either coherence or streaming. In such instances, topographically-restricted prediction errors may be continually generated in tinnitus, along with top-down feedback.

Generally, individuals with tinnitus display impaired performance compared to non-tinnitus persons on various formal working memory and/or attention tasks [13], deficits in learning and serial encoding [14], reaction time [15], decreased autobiographical memory [16,17], and diminished performance on Stroop tasks measuring selective attention [18]. The general depletion of resources theory [19] accounts for this observation as that cognitive resources are directed towards the tinnitus signal, there is less cognitive reserve left to carry out the tasks. Although the evidence is not conclusive that cognition is poorer amongst persons with tinnitus, some tinnitus distress likely arises due to the effects of cognitive deficits (working memory and attention limitations), emotional processing bias, and negative appraisal [20]. 

Sound therapy is a common tinnitus treatment component; conventionally sound is used to cover or compete with the tinnitus. The mechanisms by which sound therapy operates in the short- and long-term are unclear; it may reduce tinnitus through its presence, context, reaction, and potentially through adaptation mechanisms [21]. This study employs a novel sound that is cross-faded (“morphed”) from resembling the person’s tinnitus (the tinnitus avatar) to a nature sound that shares some of the features of the avatar but is a real “everyday” sound. 

This research addresses Feldmann’s [10] hypothesis that if tinnitus could be made more real, the ability to adapt to it would be easier. Here we undertake a preliminary evaluation to test whether perceptual training, attempting to reduce tinnitus’ discrepancy with real-world auditory objects, may alleviate tinnitus severity. Specific characteristics of sound delineate perceptual categories (which determine how sets of things are conceptualized). Categories can be formed by a set of things (e.g., animals) or based on the degree (e.g., small or big). 

Categorical perception can be inborn (e.g., basic color discrimination such as red or yellow) [22,23,24,25]. However, in the areas of uncertainty, context, contrast cues as well as individual thresholds play a role, and it is possible to induce by learning (e.g., whether shades of orange fall into red or yellow) [26,27,28,29]. A categorical expansion occurs when the classifications and boundaries for the category become broader, encompassing a larger set of objects; categorical compression is the narrowing of boundaries such that the “edge lines” are closer together [30]. Bergman [31] further deduces two dimensions for the categorization of sound: Perceptual and emotional. The latter involves grouping items together based on affective response. The uncanny valley hypothesis (UVH) refers to the psychological phenomenon whereby perceptual stimuli that are at the ambiguous boundary on a continuum in “human” and “non-human” categories give rise to a strong, negative affective response [32]. This may be related to the case of tinnitus, which is by nature a “human” sound but not one which the individual can often identify with or place entirely as environmental as it does not follow certain characteristics present in the external sound. This ambiguity in categorization may be one contributory factor towards tinnitus distress. 

Auditory object perceptual training may be one possible solution if this is where the problem lies. Perceptual characteristics or interpretation of tinnitus can potentially be modified, thus it is regarded as a distinct auditory object, i.e., to facilitate the completion of auditory analysis processing of tinnitus. This is anticipated to also facilitate a reduction in tinnitus distress and emotional affect. Nature/environmental sounds have been used extensively in sound therapy paradigms [33,34]; these seem to show benefit by masking tinnitus as well as by providing an alternative sound that is more pleasant to listen to, providing psychological benefit or a ‘context of sound’ effect [21]. This study is a novel exploration of underlying psychoacoustic correlates of tinnitus and networks associated with tinnitus auditory streaming and how malleable these are to change with targeted intervention, but builds on extensive ground work examining attention effects on tinnitus and auditory object streaming, negative emotions involved in tinnitus distress, and the role psychological variables play in sound therapy [12,35,36,37,38]. The findings from these studies can inform understanding and further development of work related to higher-level, psychoacoustic models of tinnitus [7,8].

This study is a feasibility trial with the purpose of conceptualizing parameters for auditory object perceptual training and underlying neural correlates of training effects, which can serve as a foundation for future clinical trials on a larger sample of participants. A tinnitus avatar is first generated that is identical to individual tinnitus. This sound is then morphed slowly over time until it is identical to an environmental sound. Within the field of virtual reality, one study has attempted to synthesize an auditory replica of tinnitus. However, this relied purely on auditory thresholds, tinnitus pitch, and participant preference judgments [39]. 

Electroencephalography (EEG) recordings were undertaken through the trial (before, during, and after the sound training). EEG measures the electrical activity of the brain with excellent temporal resolution across the scalp; and is relatively inexpensive. However, maximizing the utility of EEG data requires the integration of both temporal and spatial characteristics. We, therefore, applied a sophisticated analytical tool based on one of the most promising trends of artificial intelligence (AI) techniques, called deep brain-inspired spiking neural networks (SNN) architecture. The brain-inspired SNN model can incrementally learn from brain dynamics gathered over time in a 3D space of artificial neurons and capture meaningful patterns from brain data [40,41]. This will lead to a better understanding of the brain processes and changes that may be involved during the sound training. 

It is hypothesized that (1) the tinnitus avatar generated using the parameters (pitch, bandwidth, spatial location) would be an appropriate replication to individual tinnitus as judged using likeness rating scales, and the sound would not be unpleasant enough to evoke negative reactions upon administration as judged through pleasantness rating scales; the environmental sounds selected for this trial will have an appropriately high resemblance to individual tinnitus, and the sound would be more pleasant to listen to as judged through similar rating scales; (2) sound therapy customized to shift perceptual judgments of tinnitus towards a real auditory object (environmental sounds) will result in reduced tinnitus perception and distress, specific training times and type of object morphing parameters will show increased benefit than others; and (3) perceptual changes with therapy may correlate with changes in activation of modeled tinnitus neural networks, particularly relating to attention and discriminatory judgments (e.g., dorsal attention network, precentral gyrus, ventral anterior network). 

## 2. Materials and Methods

This study was approved by the University of Auckland Human Participants Ethics Committee. All subjects gave written informed consent in accordance with the Declaration of Helsinki.

### 2.1. Participant Eligibility and Recruitment

**Figure 1:**
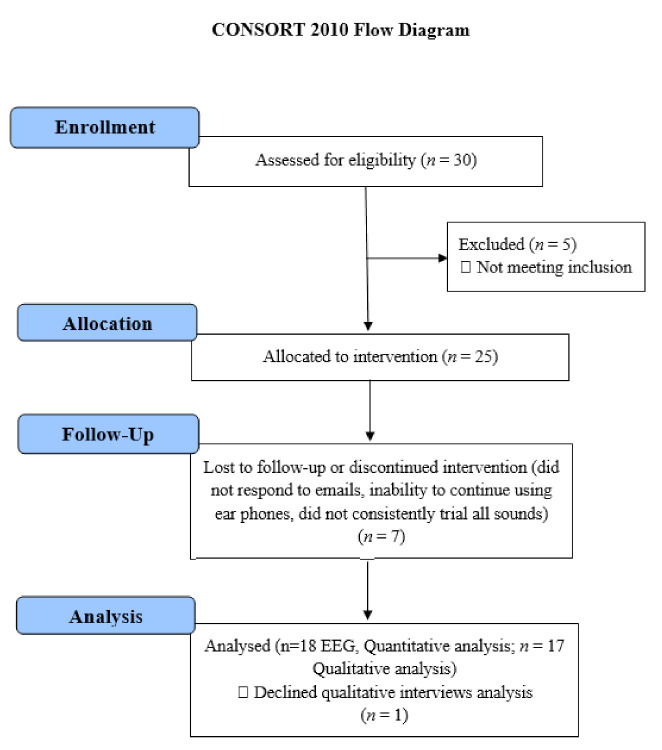
Consolidated Standards of Reporting Trials (CONSORT) reporting of the study.

**Figure 2:**
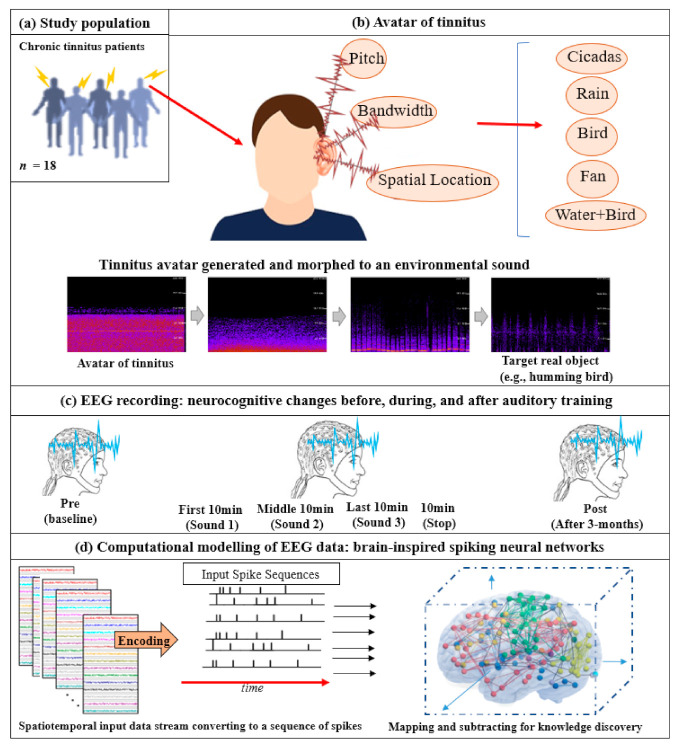
The study protocol: Diagram of data collection across (**a**) 18 chronic tinnitus patients that underwent auditory training through (**b**) depicting parameters (pitch, bandwidth, and spatial location) used to determine tinnitus avatar; and conceptualization of gradual morphing of high-pitched tinnitus from its current perceptual characteristics (spectral change is illustrated in this example) to that matching a real target auditory object (a humming bird is used in this example); (**c**) spatiotemporal brain data measured before, during, and after the auditory training; (**d**) illustration of the SNN-based methodology, containing: EEG encoding into spike sequences and computational modeling of data into a 3D space of artificial neurons.

Email invitations were sent out to the University of Auckland Tinnitus Research Volunteers Database. A participant information sheet was provided to participants that outlined the background and aims of the trial and details of measurements to be taken at various appointments. The inclusion criteria for the study were: Adults aged above 18 years residing in the Auckland region (NZ), constant tinnitus (that did not regularly fluctuate in its psychophysical properties and of minimum 6 months duration), a minimum score of 21 on the Tinnitus Functional Index (TFI) (this cut-off score was calculated based on convergent validity results between TFI mean scores and response levels of a tinnitus global severity item; a score of 21 delineated individuals who considered their tinnitus as problematic from those who did not view tinnitus as a problem), normal middle ear function, and a maximum of a moderate–severe degree of hearing loss (less than 90 dB loss on average across the frequency range of 125–8000 Hz). Eighteen participants (8 females, 10 males, mean age = 61.6 years, SD = 14.8, range 21–74) from the University of Auckland Tinnitus Research Volunteer Database met the inclusion criteria, were recruited, and completed the trial. Five participants did not meet the criteria and were excluded. Seven participants met the inclusion criteria and commenced the trial but were lost to follow-up (did not respond to emails) or discontinued intervention (inability to continue using earphones, did not consistently trial all sounds). In such cases, both behavioral and electrophysiological data were not usable. The same participants took part in Part 1 and Part 2. of the study. The CONSORT reporting flowchart, study protocol, and the SNN-based methodology are depicted graphically in Figure 1 and Figure 2. In the following, the study protocols and the experiments will be discussed. 

### 2.2. Part 1. Development of Tinnitus Avatar, Real Auditory Objects Library, and Acute Electroencephalography (EEG) Stepped Morphing Measurements

#### 2.2.1. Tinnitus Avatar

All participants had their auditory thresholds initially measured with pure-tone audiometry in a sound-proof booth using the modified Hughson Westlake procedure in a sound booth (ISO 8253-1) with a GSI 61 audiometer. Air conduction thresholds were recorded for 125–8000 Hz using insert earphones (ER-3A) or supra-aural (TDH-39P) transducers. Where a hearing loss was found, bone conduction testing at 500, 1000, 2000, and 4000 Hz was performed using a Radioear B-71 bone conductor transducer to ascertain conductive or sensorineural hearing loss. Air conduction thresholds at extended high frequencies (9000, 10,000, 12,500, 14,000, and 16,000 Hz) were tested using Sennheiser HDA 200 circumaural headphones. Immittance testing consisted of tympanometry. 

Tinnitus psychoacoustic testing software (©The University of Auckland) installed on a DELL Latitude E6400 laptop computer was run with participants in the sound-proof booth using circumaural Sennheiser HDA 200 transducers. The pitch of tinnitus was measured with the participant responding to a 2-alternative, forced-choice method of pitch matching presented at 15 dB SL (Sensation Level), also checking for octave confusion. Spatial location of tinnitus was matched through a virtual 3D space interface available in the testing software (using Head-Related Transfer Function). Loudness matching in dB SL was performed in two steps: (1) The threshold level was determined for the pitch of tinnitus as per standard audiometry; (2) intensity was increased from threshold until the participant indicated a good match to the subjective loudness of their tinnitus. Minimum masking level (MML) in dB SL to 1/3 octave band noise was obtained by a similar method, and participants were instructed to indicate when the sound was loud enough to cover their tinnitus.

Following this, a tinnitus ‘avatar’ was generated for each participant using Adobe Audition software. As each parameter was mapped, participants provided real-time ratings on a scale of 1 (does not sound anything like my tinnitus) to 10 (is an exact replication of my tinnitus); rating scores alongside question prompts from the researcher together helped determine the final parameter match. The first psychoacoustic parameter mapped was Pitch. A tonal sound was generated, and the pitch was increased in half-octave steps between 250–16,000 Hz, until participants matched the pitch as being the most similar to their tinnitus. Following this, a 3D audio plugin function was used on the software to change the perceived spatial location of the tone in space in 30° steps, from left to right on the horizontal plane, as well as from bottom to top of the head in perceived elevation. Participants indicated when the tone overlapped with their tinnitus in 3D space. For central tinnitus or tinnitus that was perceived as being equal in both ears, the standard stereo tonal sound was sufficient for some participants to overlap with their tinnitus perceived location. The third parameter matched was the Bandwidth of the sound, which was increased as follows: 0, 1/24, 1/12, 1/6, 1/3, 1/2, 1, 2, 3, 4 octaves wide; participants indicated when the sound quality was identical to their tinnitus. 

The corresponding sound generated combining these 3 parameters simulated a current ‘avatar’ of tinnitus as an auditory object. Participants were given the option of fine-tuning the sound using ± 2 step options along any of the parameters they felt could be optimized a bit more. The final tinnitus avatar was rated on a likeness scale of 1 (does not sound anything like my tinnitus) to 10 (is an exact replication of my tinnitus), and on a pleasantness scale of 1 (highly unpleasant) to 10 (highly pleasant). If, during the task, participants started to perceive more than one tinnitus sound, the most prominent and/or debilitating tinnitus sound was asked to be replicated. All sounds were played at a comfortable listening level, and the final tinnitus avatar sound was adjusted for individual hearing thresholds using frequency channel-volume adjustments on Adobe Audition. An appropriate tinnitus avatar was pre-determined by the authors to constitute a likeness rating of 7 or greater (on the 1–10 scale), as well as an affective rating of at least 4 (on the 1–10 scale).

#### 2.2.2. Real Auditory Objects Library

A library consisting of 5 real, neutral auditory objects was compiled using royalty-free sources. The environmental sounds were: Cicadas, Fan, Water Sound/Rain, Birds and Water + Birds, and were selected based on common reports of similarities to tinnitus, as well as differing frequency and bandwidth characteristics. All files were 1 hour long in duration and adjusted for volume intensity to match each other in long-term loudness level spectrum. Each participant matched their tinnitus to one of these environmental sounds for perceptual training using a tournament selection procedure, based on Audiologist suggestions of similarity to tinnitus, emotional ratings of the auditory object, and participant preference.

#### 2.2.3. Acute EEG Stepped Morphing

An ‘acute’ or fast stepped morphing file was generated using the cross-fade function on Adobe Audition, such that the tinnitus avatar morphed into the matched environmental sound over a period of 1 hour. In the first 10 minutes, the tinnitus avatar had predominance (90% intensity ratio), and this decreased steadily such that in the last 10 minutes of the file, the tinnitus avatar had little presence (10% intensity ratio) compared to the environmental sound. 

Participants had EEG recordings conducted while sitting in quiet (10-min baseline/Pre; ear phones in, no sound playing, watching a grey cross presented in the center of a computer monitor screen placed 1.5 m away) and then while listening to this 1 hour stepped morphing file at 3 time points: During the first 10 min of the clip, in the middle 10 min of the clip (0:25:00 to 0:35:00), and in the last 10 min of the clip. A quiet 10-min EEG was taken immediately after the participant stopped listening to the sound file at the end of the hour (Post). Recordings were conducted in a dimly lit sound-attenuating room (shielded environment) using 66 active surface electrodes (Biosemi ActiveTwo system) placed on the scalp according to the international 10/20 system array through attachment to an appropriately sized Biosemi 64 electrode head cap with SignaGel electrode gel. All sound stimuli were presented via E.A.R Tone 3A Insert earphones (Etymotic research) and controlled using Presentation 17^®^ Software run from a desktop computer. EEG signals were recorded continuously at a sampling rate of 8192 Hz and down sampled to 256 Hz for analysis.

### 2.3. Part 2. Three-Month Feasibility Trial

#### Chronic Sound Stepped Morphing

The purpose of the 3-month feasibility trial was to ascertain longer-term, chronic effects of tinnitus auditory object perceptual training. Four versions of sound files were created for each participant, with the sound therapy stimulus being between each version to facilitate shifts towards the selected environmental sound. For the first 2 weeks of the trial, the 1-hour tinnitus ‘avatar’ sound file was played in order for the participant to learn to associate this external sound with their own tinnitus. Between 2 weeks and 1 month of the trial, the sound morphed from 100% tinnitus avatar to 50% tinnitus avatar/50% environmental sound intensity composition during the 1-hour file. Between 1 month and 2 months of the trial, the sound morphed from 50% tinnitus avatar/50% environmental sound to 10% tinnitus avatar/90% environmental sound during the 1-hour file. For the last version, between 2 and 3 months of the trial, the sound morphed from 10% tinnitus avatar/90% environmental sound to 100% environmental sound during the 1-hour file. As in Part 1, sounds were adjusted for hearing thresholds as well as participant desired listening levels.

Participants were fitted bilaterally with take-home ear-level MP3 players to deliver the stepped morphing sounds and were instructed to use the devices for 1 hour per day in quiet environments. The 4 versions of sound were adjusted for hearing thresholds as well as participant comfort using Audacity software. The first sound version was loaded onto the MP3 device at the beginning of the trial; subsequent versions were emailed to participants at the appropriate time points (2 weeks, 1 month, and 2 months) for them to download onto the device and listen. At these time points, tinnitus and well-being questionnaires and qualitative questions (described under Outcome Measurements) were also administered by email to monitor changes over time. If participants were comfortable having the versions manually loaded on the device for them, they were encouraged to come into the clinic to have this done. At any time, participants were able to adjust the volume of the sound file according to comfort. 

After 3 months, participants came back for a final appointment. Tinnitus and well-being questionnaires were administered, and tinnitus psychoacoustic matching of Pitch, Spatial Location, LLM, and MML was conducted again using the tinnitus testing software. An end-of-trial qualitative interview was conducted, informational counseling was provided and any questions or concerns were addressed. Participants who wanted to carry on listening to the sounds were encouraged to do so using their personal devices. A 10-min EEG in quiet (3-month Post; earphones in, no sound playing, watching a grey cross presented in the center of a computer monitor screen placed 1.5m away) was obtained in an identical set-up to the acute EEG stepped morphing procedure.

### 2.4. Outcome Measures: Statistical Analysis and Computational Modeling of Data

#### 2.4.1. Questionnaires

Tinnitus Functional Index (TFI) [42] scores were used to assess changes in tinnitus impact on life. The TFI used an 11-point numeric scale (from 0 or 0% indicating no tinnitus problem to 10 or 100% indicating a very big problem) and had 8 domains assessed: Intrusiveness, sense of control, cognitive, sleep, auditory, relaxation, quality of life, and emotional impact of tinnitus. This has been validated for test-retest reliability and has high responsiveness to treatment-related change and internal consistency in New Zealand [43]. Numeric rating scales were used to measure tinnitus perception along 5 dimensions: How strong, intrusive, uncomfortable, unpleasant the tinnitus signal was; how easy it was to ignore the tinnitus signal. The Positive and Negative Affect Schedule (PANAS) [44] measured the extent to which positive and negative emotional states were experienced by an individual over the period of the previous week. The 42-item Depression, Anxiety, and Stress Scale (DASS) [45] was used to measure levels of psychological affective symptoms of anxiety, depression, and stress. All questionnaires were administered at the beginning of the trial, at 2 weeks, 1 month, 2 months, and at 3 months following the start of the training. Data were also normally distributed; thus, parametric statistical tests were used. A repeated-measures Analysis of Variance (ANOVA) was run for all 4 questionnaire outcome measures (overall scores and subscales were relevant) to test for significant changes over time (baseline, 2 weeks, 1 month, 2 months, and 3 months) and between-subjects factors of intervention sound (Cicadas, Fan, Water Sound/Rain, Bird, Water + Bird) and tinnitus location (Pre/Post) introduced to examine for intervention sound type and tinnitus laterality effects. Where Mauchly’s test of Sphericity was significant, Greenhouse–Geisser corrections were used. 

#### 2.4.2. Psychoacoustic Tinnitus Characteristics

Tinnitus psychoacoustic characteristics of pitch, spatial location, LLM, and MML were measured at the beginning of the trial and at the end of the trial (3 months). Descriptive analysis of pitch and spatial location changes were conducted and a paired samples t-test was run to test for significant changes in LLM and MML at baseline and after 3 months of sound use.

#### 2.4.3. Qualitative Interviews

Patient-reported ratings of tinnitus and any incidental observations were asked via email and recorded at 2 weeks, 1 month, 2 months, and at 3 months of the trial (see Appendix A for list of questions asked and response excerpts from participants). The framework method [46] was used to analyze the responses, consisting of 5 steps: Familiarization, identification of a thematic framework, indexing, charting, and mapping and interpretation. Common themes were identified in the individual’s responses (by author MD), and in the charting phase, the data were rearranged according to theme. In the mapping and interpretative stages, the charted data were compared and contrasted to identify patterns within the data (MD in consultation with GDS). Quotations from participants and their thematic analysis were included in the results following standard practice in qualitative methodology [47,48].

#### 2.4.4. Electroencephalography (EEG) 

EEG recordings obtained were used to examine preliminary acute effects of stepped morphing by comparing brain activity prior to morphing sound presentation, brain activity during the first 10-min of the file with tinnitus avatar 90%: Environmental sound 10% intensity composition (Sound 1), during the middle 10 min of the file [50% tinnitus avatar: 50% environmental sound] (Sound 2), during the last 10 min of the file [10% tinnitus avatar: 90% environmental sound] (Sound 3), 10 min immediately following completion of sound presentation (Post) and brain activity in quiet but following 3-month feasibility trial administration (3-month Post). The last measurement was a measure of anticipated chronic or sustained longer-term changes following perceptual auditory training.

#### 2.4.5. Electroencephalography (EEG) Modeling and Analysis Using Brain-Inspired Spiking Neural Networks Architecture

The brain data analysis in this study were based on one of the most promising trends of Artificial Neural Networks (ANN) tools, called Spiking Neural Networks (SNN) method. SNN-based methodology has been developed as a neurobiologically-plausible computational architecture that incorporates both spatial and temporal EEG data characteristics into one computation. In an SNN model, an artificial spiking *neuron* is an information processing element facilitated with a learning algorithm that extracts the relationship between the streaming data variables over time [49]. That is, spiking *neurons* are connected by synapses, where the learning patterns are memorized. Compared with conventional machine learning methods, SNN models integrate the notion of time into the computation and thus are considered to be superior in biological plausibility in neural networks compared to previous models that do not account for temporal dynamics. Thus, SNNs are recognized as appropriate models for processing Spatio-Temporal Brain Data (STBD) [50,51,52,53,54]. 

The SNN architecture includes several modules, here SNN was used for mapping, learning, visualizing, and to model the EEG data that are measured at “before”, “during” and “after” the sound training to investigate the neural tinnitus networks changes.

The experiment protocol and the SNN-based methodology for this case study is shown graphically in Figure 2. It is constituted of the following steps:

- Data encoding: In the first phase of data modeling, the real-value EEG time series was encoded to trains of spikes using an appropriate encoding method [55]. As shown by Petró et al., the Step Forward [55] algorithm best captured the characteristics of the original signals. The SF algorithm employed a fixed threshold and a moving baseline, which was adjusted after each spike was created. Using this technique, if the upward change in a signal’s amplitude from the baseline was more than the threshold at a certain time, a positive spike was produced. Conversely, negative spikes were created if the amplitude diminutions from the baseline below the defined threshold. When none of these cases occurred, no spikes were generated. The generated spike trains embody changed in the STBD that exceeded a threshold SFthr. Since this threshold was dependent on the signal amplitude, it was optimized before the encoding procedure to more accurately capture the signal changes. Figure 2d demonstrates an instance of encoded EEG signals into positive and negative spike trains generated from the raw EEG data.
- Mapping: Figure 2d further illustrates that a model was then pre-structured to represent the functional and structural information of the brain processes measured by spatiotemporal data. The STBD data samples were mapped spatially into 3D artificial neural space where the spatial information of brain areas was topologically preserved concerning the (x, y, z) coordinates as positioned in the Talairach brain atlas [56]. In the SNN model, after defining a biologically plausible 3D SNN, data were initialized with a Small-World Connectivity rule (SWC) [57] that defined a probability by which a neuron *i* can be linked to a neuron *j* with respect to their internal distance, the greater the distance between *i* and *j*, the smaller the connection probability. The generated initial connections were adapted during the unsupervised learning process, which takes into account the temporal dynamics of input data [58].
- Learning: The SNN model used an unsupervised learning algorithm, called Spike Time-Dependent Plasticity (STDP), which allows the model to learn the spatiotemporal relationships in the input spikes [50]. This learning process modifies the neural connection strength with respect to the timing of pre to postsynaptic neurons. The connections between neurons were updated dynamically at each time point of the input data (e.g., at a millisecond scale), resulting in deep trajectories of connectivity learnt in the 3D SNN structure. Throughout the STDP learning process, if every neuron’s potential passed an activation threshold in time t, then it produced an output spike. The spike was then transferred to other neurons linked to it. This neuron likewise kept receiving spikes over time and, after passing a threshold, fired. In this way, spikes were propagated inside the SNN model during the STDP learning and the ‘hidden’ spatiotemporal relationships between the data variables were captured in the shape of neural connectivity and the weights of these connections. The connection weights can then be visualized [59,60,61].
- Pattern visualization: Different models were then trained with the EEG data extracted from different brain mental states, e.g., before, during, and after sound training across participants, thus different models were produced thus they could be further compared and analyzed. The models were subtracted to understand the difference between the two states as a result of different brain activities.

## 3. Results

### 3.1. Participant Hearing and Tinnitus Characteristics

**Figure 3:**
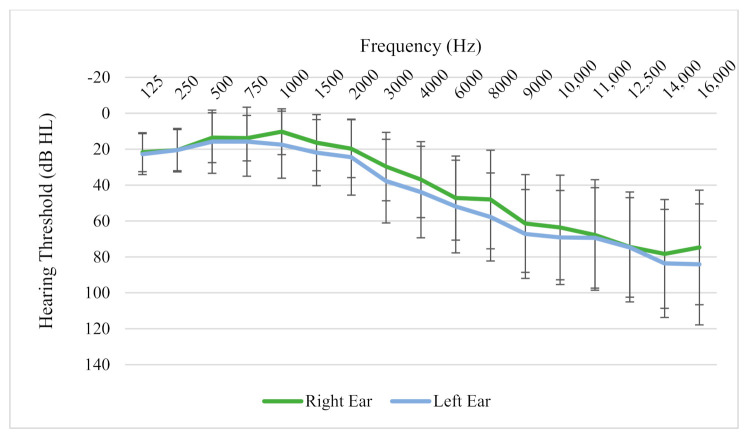
Average hearing thresholds (dB HL) of participants across frequencies for the Right and Left ear. Error bars represent ± 1 SD.

Average hearing thresholds of participants showed normal low-frequency hearing, sloping gently from 2000 Hz onwards to become a moderate hearing loss at 8000 Hz, then sloping further in the extended high frequencies to moderately severe/profound by 16,000 Hz (Figure 3). Hearing thresholds were fairly symmetrical between the two ears. The mean Tinnitus Functional Index (TFI) score of participants at the beginning of the trial was 30.4 (SD = 4.0). All participants had experienced chronic bothersome tinnitus for a minimum of 1 year with an average length of time since tinnitus onset of 20.5 years (SD = 20.4, ranging from 1–60 years). Twenty-two percent of the participants described tinnitus quality as cricket sounds, 50% as tonal, 17% as noise, and 11% described their tinnitus as a combination of sounds or ‘other’. Location of the tinnitus was: In the right ear (22%), left ear (28%), or in both ears equally (50%) for participants (Figure 3). The measured tinnitus pitch of participants ranged from 561 Hz to 10,000 Hz, and there was no clustering observed around any particular pitch match. Sixty-one percent of participants had not used any form of tinnitus treatment in the past, 11% had tried one treatment, and 28% had tried more than one treatment. Five out of the 18 participants (27%) wore hearing aids bilaterally; 2 participants (11%) wore hearing aids in one ear only. When asked whether loud sounds tended to make their tinnitus worse, 44% responded that it did exacerbate it, 56% responded no. Sixty-one percent of participants felt that their tinnitus was reduced by music or by certain types of nature sounds (such as the noise of a waterfall, running shower water, etc.), 6% felt it was not, and the remaining 33% did not know.

### 3.2. The Tinnitus Avatar

The tinnitus avatar generated by participants was rated by participants to be a good match to individuals’ tinnitus (average likeness rating to tinnitus of 7.75/10, where 1 = did not sound like tinnitus at all; 10 = identical to tinnitus) and was not deemed by participants as being too unpleasant to listen to (average pleasantness rating of 5.78/10, where 1 = is not pleasant at all; 10 = is highly pleasant). The environmental sounds used in the study (Cicadas, Fan, Water Sound/Rain, Birds, Water + Bird) were a good alternative match to tinnitus (average likeness rating to tinnitus of 7.28/10) and were largely pleasant to listen to (8.47/10).

### 3.3. Questionnaires Outcomes

#### 3.3.1. Tinnitus Functional Index (TFI)

**Figure 4:**
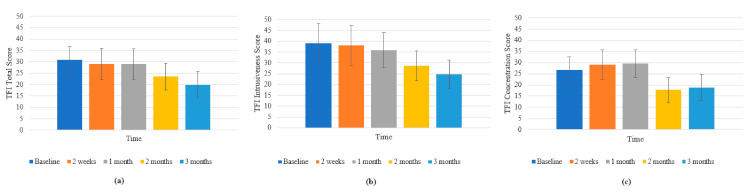
(**a**) Changes in TFI Total scores across time; (**b**) changes in TFI Intrusiveness scores across time; (**c**) changes in TFI Concentration scores across time. Error bars represent ±1 SD.

There was a significant effect of time on the total score of the Tinnitus Functional Index (F (2.164, 23.8) = 3.366, *p* = 0.048; *η*^2^ = 0.234). The TFI total score at 3 months (M = 19.940, SD = 5.771) was significantly lower than at baseline (M = 30.910, SD = 5.857; *p* = 0.047) (Figure 4a). When broken down into subscales, there was a significant effect of time on the TFI Intrusiveness subscale (F (2.311, 25.423) = 5.519, *p* = 0.008; *η*^2^ = 0.334). TFI Intrusiveness at 3 months (M = 24.667, SD = 6.543) and at 2 months (M = 28.667, SD = 6.893; *p* = 0.029) was significantly lower than at baseline (M = 39.083, SD = 8.988; *p* = 0.013) (Figure 4b).There was a significant effect of time on the TFI Concentration subscale (F (4, 29.536) = 3.798, *p* = 0.01; *η*^2^ = 0.257) with the score at 2 months (M = 17.833, SD = 5.482) being significantly lower than at 1 month (M = 29.5, SD = 6.217; *p* = 0.032) (Figure 4c).There was a marginally significant effect of time on the TFI Emotion subscale (F (1.914, 21.052) =3.402, *p* = 0.054; *η*^2^ = 0.236). There was also a significant interaction effect of type of intervention sound and time on the TFI Concentration subscale (F (12, 33) = 4.625, *p* < 0.001; *η*^2^ = 0.627). At 2 months, there was a significant difference in TFI concentration scores of participants who had the Water and Bird sound (M = −36.667, SD = 11.78) compared to: Cicadas sound (M = −1.667, SD = 8.33; *p* = 0.034); Fan sound (M = 3.333, SD = 11.78; *p* = 0.035); Water Sound/Rain (M = −6.25, SD = 4.165; *p* = 0.033) and Birds sound (M = −3.333, SD = 5.89; *p* = 0.028).The Water and Bird sound seemed to result in a much greater decrease in TFI concentration scores in comparison to the other sounds.

**Figure 5:**
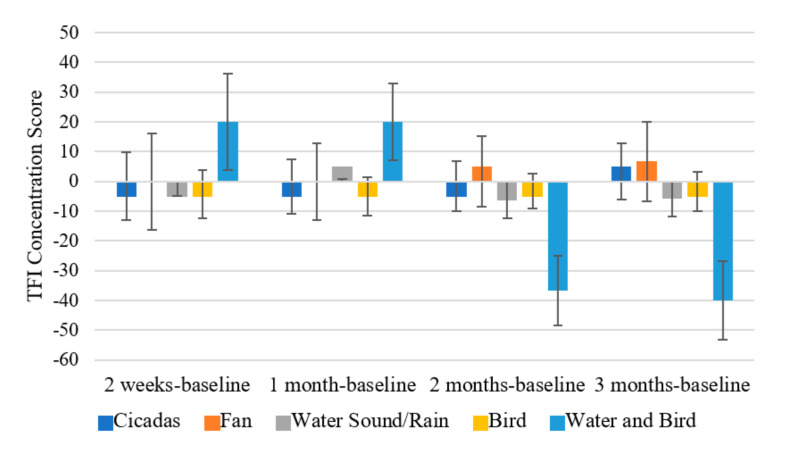
Changes in TFI Concentration scores across time by Intervention sound type. Error bars represent ± 1 SD.

At 3 months, this effect persisted with Water and Bird (M = −40, SD = 13.314) having a much lower TFI concentration score compared to: Cicadas (M = 3.333, SD = 9.415; *p* = 0.022), Fan (M = 6.667, SD = 13.314; *p* = 0.031), Water Sound/Rain (M = −5.833, SD = 4.707; *p* = 0.034) and Birds (M = −3.333, SD = 6.657; *p* = 0.032) (Figure 5). 

#### 3.3.2. Tinnitus Severity Numeric Rating Scales

**Figure 6:**
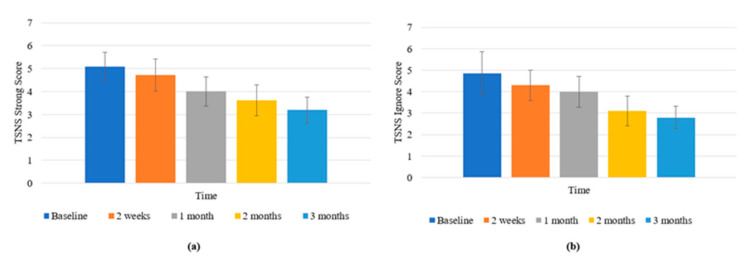
(**a**) Changes in TSNS Strong scores across time; (**b**) changes in TSNS Ignore scores across time. Error bars represent ±1 SD.

There was a significant effect of time on the Strong rating scale (F (4, 44) = 6.178, *p* <0.001; *η*^2^ = 0.360). The score at 2 months (M = 3.625, SD = 0.681) was significantly lower than at baseline (M = 5.1, SD = 0.622; *p* = 0.013); and at 3 months (M = 3.2, SD = 0.565) was significantly lower than baseline (*p* = 0.006) (Figure 6a).

There was a significant effect of time on the Ignore rating scale (F (1.89,20.786) = 4.036, *p* = 0.035; *η*^2^ = 0.268). The score at 3 months (M = 3.1, SD = 0.69) was significantly lower than at 2 weeks (M = 4.3, SD = 0.71; *p* = 0.027) (Figure 6b). There was a marginally significant effect of time on the Overall rating scale (F (2.194, 24.129) = 3.176, *p* = 0.056; *η*^2^ = 0.224). 

#### 3.3.3. Depression, Anxiety, and Stress Scale (DASS)

There were no significant effects, main effects of time, or interaction effects of intervention sound type on DASS scores for Depression, Anxiety, or Stress. 

**Figure 7:**
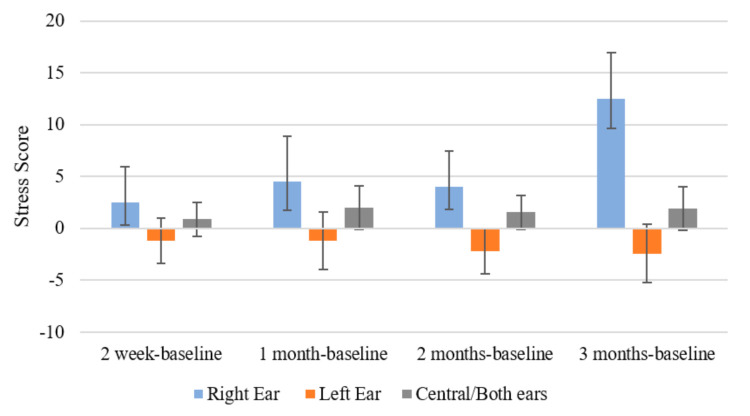
Changes in DASS Stress scores across time by Tinnitus Location. Error bars represent ± 1 SD.

There was a significant interaction effect of time and initial location of the tinnitus (F (3.333, 21.662) = 4.951, *p* = 0.008; *η*^2^ = 0.432) for DASS Stress. Those with right-sided tinnitus at the beginning of the trial experienced a greater increase in stress scores at 3 months (M = 12.5, SD = 4.457) compared to participants with left-sided tinnitus (M = 2.4, SD = 2.819; *p* = 0.014). A marginally significant difference was present between stress scores at 3 months compared to baseline for right-sided tinnitus and central tinnitus (M = 1.889, SD = 2.101; *p* = 0.051) (Figure 7). 

#### 3.3.4. Positive and Negative Affect Schedule (PANAS)

**Figure 8:**
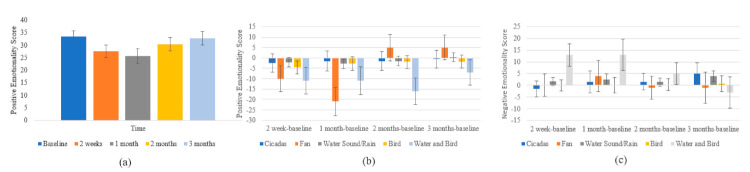
(**a**) Changes in Positive Emotionality PANAS scores across time; (**b**) changes in Positive Emotionality PANAS scores across time by intervention sound type; and (**c**) changes in Negative Emotionality PANAS scores across time by intervention sound type. Error bars represent ±1 SD.3.3.5 Psychoacoustic tinnitus characteristic changes.

There was a significant effect of time on the Positive Emotionality subscale of the PANAS (F (1.9, 20.895) = 9.047, *p* = 0.002; *η*^2^ = 0.451). The score at 3 months (M = 32.7, SD = 2.684) was significantly higher than at 2 weeks (M = 27.45, SD = 2.451; *p* = 0.023); at 2 months (M = 30.275, SD = 2.731) was significantly higher than at 1 month (M = 25.65, SD = 2.842; *p* = 0.002); and the score at 3 months was significantly higher than at 1 month (*p* < 0.001) (Figure 8a). 

There was also a significant interaction effect of type of intervention sound and time on the Positive Emotionality subscale of the PANAS (F (8.872, 24.399) =8.105, *p* <0.001; η^2^ =0.747). At 1 month, participants who had the Fan sound seemed to experience a much greater decrease in positive emotionality (M = −21, SD = 6.689) compared to: Cicadas (M = −1.5, SD = 4.73; *p* = 0.036); Water Sound/Rain (M = −2.875, SD = 2.365; *p* = 0.027); and Birds (−2.75, SD = 3.344; *p* = 0.033). At 2 months, participants using the Water and Bird sound (M = −16, SD = 6.342) seemed to have a much greater decrease in positive emotionality compared to Fan (M = 5, SD = 6.342; *p* = 0.039) (Figure 8b). 

There was a significant interaction effect of type of intervention sound and time on the Negative Emotionality subscale of the PANAS as well (F (5.913, 16.26) = 3.111, *p* = 0.032; η^2^ = 0.531). At 2 weeks, participants with the Water and Bird (M = 13, SD = 4.771) sound had significantly greater negative emotion scores compared to: Cicadas (M = −1.5, SD = 3.374; *p* = 0.030); Water Sound/Rain (M = 1.625, SD = 1.687; *p* = 0.046) and Birds (M = 0, SD = 2.2385; *p* = 0.033) (Figure 8c). 

**Figure 9:**
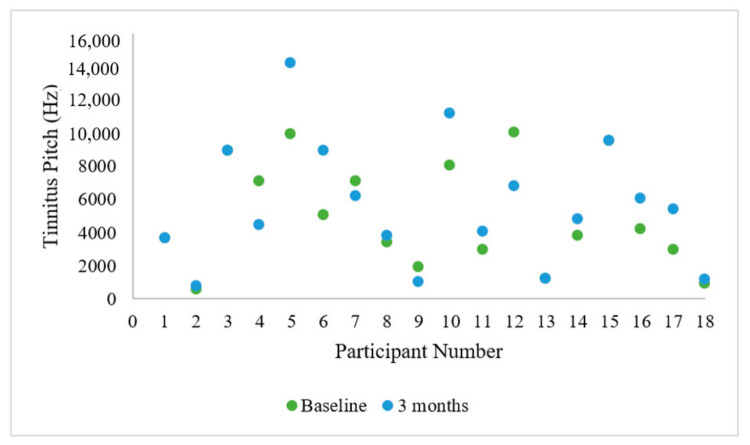
Tinnitus pitch match for participants in Hz at baseline and at 3 months following the end of the feasibility trial.

There was a significant reduction in MML at 3 months (M = 10.71, SD = 9.56) compared to baseline (M = 16.82, SD = 11.98; *t* (16) = 2.177, *p* = 0.045). Three participants experienced a change in tinnitus location between the start and end of the feasibility trial (right-sided to left-sided change; central to right-sided change; central to left-sided change). Almost all participants experienced a change in their tinnitus pitch between the start and end of the trial. There was no obvious trend to the changes in pitch (Figure 9). However, it should be kept in mind that psychoacoustic tinnitus pitch tends to change in general with test-retest matching.

#### 3.3.5. Computational Modeling of EEG Data Using SNN Architecture

To perform a better analysis of brain activity changes after the auditory training, the differences between the SNN models that were trained on pre-baseline and post-sound EEG data were computed for each participant by subtracting the two trained SNN models (pre, post). This allows visualization of the changes in neural connectivity as a result of auditory training over time.

**Figure 10:**
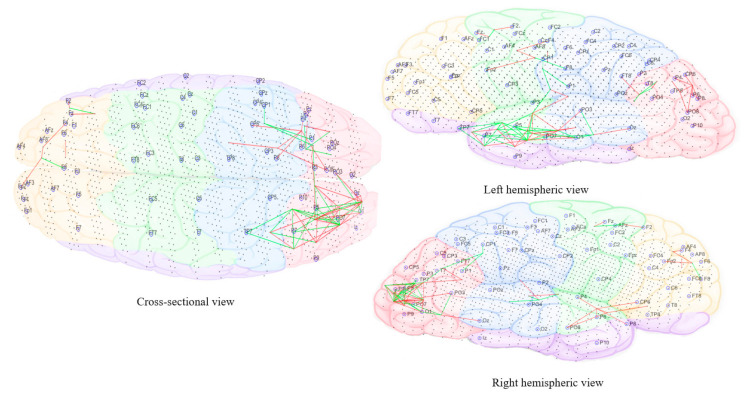
Dynamic visualization of the evolution of neuronal connectivity and spiking activity in an SNN model of 1471 spiking neurons with Talairach-based coordinates. It shows differences between the connectivity in the trained SNN models of Pre auditory training (baseline) from the first 10 min of auditory training-denoted as ‘Sound 1′. The green lines are increase connections, while the red lines are decrease connection changes. 5% of relative changes are presented.

**Figure 11:**
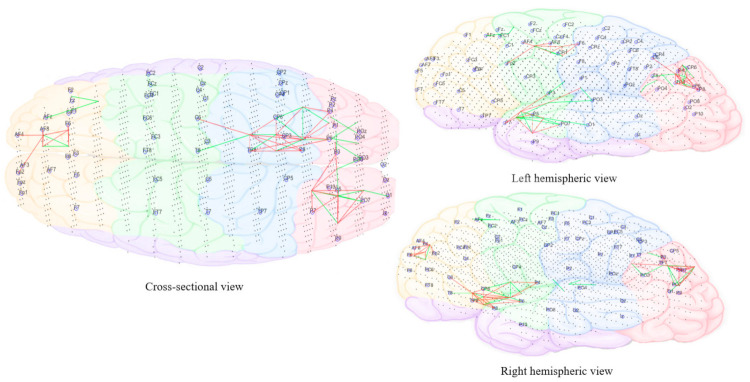
Dynamic visualization of the evolution of neuronal connectivity and spiking activity in an SNN model of 1471 spiking neurons with Talairach-based coordinates. It shows differences between the connectivity in the trained SNN models of Pre auditory training (baseline) from the middle of 10 min of auditory training-denoted as ‘Sound 2′. The green lines are increase connections, while the red lines are decrease connection changes. 5% of relative changes are presented.

**Figure 12:**
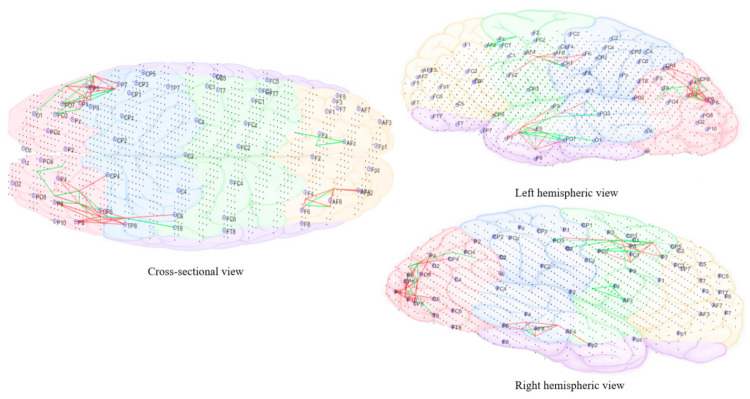
Dynamic visualization of the evolution of neuronal connectivity and spiking activity in an SNN model of 1471 spiking neurons with Talairach-based coordinates. It shows differences between the connectivity in the trained SNN models of Pre auditory training (baseline) from the last 10 min of auditory training-denoted as ‘Sound 3′. The green lines are increase connections, while the red lines are decrease connection changes. 5% of relative changes are presented.

**Figure 13:**
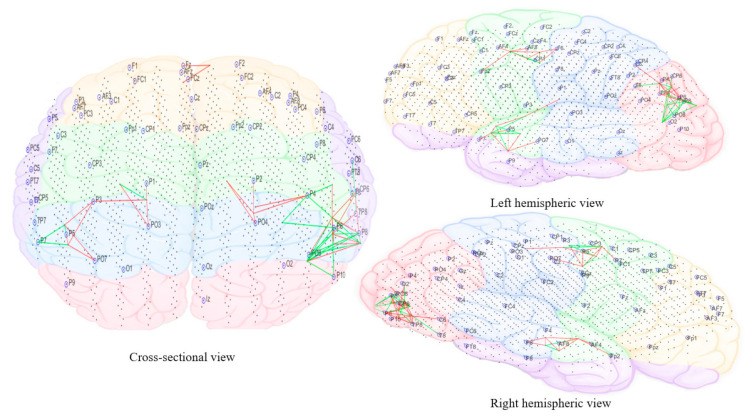
Dynamic visualization of the evolution of neuronal connectivity and spiking activity in an SNN model of 1471 spiking neurons with Talairach-based coordinates. It shows differences between the connectivity in the trained SNN models of Pre auditory training (baseline) after 10 min of stopping the auditory training-denoted as ‘Post.’ The green lines are increase connections, while the red lines are decrease connection changes. 5% of relative changes are presented.

**Figure 14:**
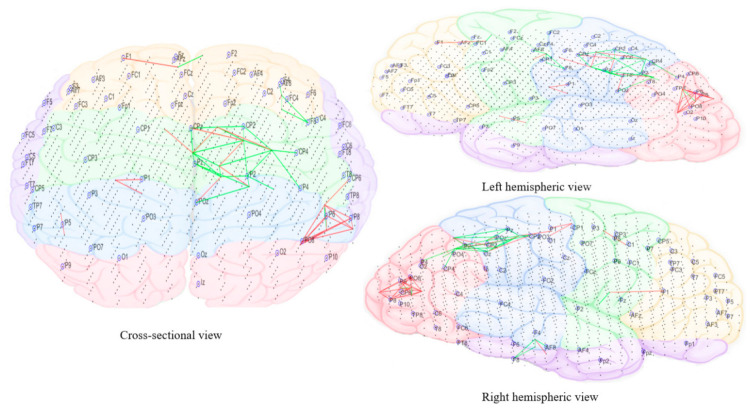
Dynamic visualization of the evolution of neuronal connectivity and spiking activity in an SNN model of 1471 spiking neurons with Talairach-based coordinates. It shows differences between the connectivity in the trained SNN models of pre-auditory training (baseline) from after 3-month post. The green lines increase connections, while the red lines are decrease connection changes. 5% of relative changes are presented.

The subtracted connectivity model was depicted in Figure 10, Figure 11, Figure 12, Figure 13 and Figure 14. It shows the involved brain areas activated in response to the sounds. The thickness of a line indicates the severity of the change. All brain activity changes described below were changes observed from the baseline/pre-measurement taken in quiet. Immediately within one minute of when the morphing sound was initially introduced (constituting of 90% resemblance to individual tinnitus, 10% resemblance to final auditory nature sound—Figure 10), there was slightly greater activity observed in right frontal electrode sites (F4, Fp2, and F2). The electrode sites correspond to BA10 (fronto-parietal cortex [62]), BA46 (medial prefrontal cortex—part of the tinnitus steady-state neural network; also, part of default mode network (DMN), which might be reduced with the presentation of sound (1) and BA08 (pre-supplementary motor cortex). In addition, there were increased changes in brain activity limited to temporo-parietal (T3, T5, and Tp7), parietal (P3), and occipital electrode sites (Po7, Po3, and O1) and showing greater left hemispheric predominance; the left hemisphere seemed to have more both increase and decrease changes while the right had only decrease changes. These electrode sites correspond to BA21 (medial temporal gyrus), BA39 (angular gyrus—spatial cognition, memory retrieval, attention), BA37 (occipital-temporal cortex; involved in visual categorization [63,64], BA40 (intra-parietal sulcus; visual processing), BA19 (cuneus; visual processing) and BA20 (inferior temporal gyrus; visual recognition). 

As the sound morphed to resemble 50% individual tinnitus/50% final auditory nature sound (Figure 11), it was possible to observe increased brain activity changes compared to the first stage sound, shifted more towards parietal, centro-parietal, and parieto-occipital electrode sites in the left hemisphere (P5, P7, P3, Cp3, and Po7). However, right hemispheric changes were also observed in temporo-parietal and parietal regions as well (P8 and Tp8). The electrode sites additionally involved correspond to BA41 (primary auditory cortex), BA02 (secondary somatosensory cortex), BA17 (primary visual cortex), and BA18 (secondary visual cortex). Ongoing activity changes in frontal electrode sites were still observable. 

In the final stage of morphing (sound is 10% individual tinnitus/90% final auditory nature sound—Figure 12), brain activity changes were localized in the left parietal (P3, P5, and P7) and right temporal (T8 and Tp8), centroparietal areas (Cp4 and Cp6) and right frontal regions (Fp2 and AF8). Direct comparison of brain activity between the final stage morphing sound and initial stage morphing sound showed greater both decrease and increase changes in parietal, parieto-occipital regions of both hemispheres predominantly corresponding to BA37 (occipito-temporal cortex), BA39 (angular gyrus), BA19 (cuneus), BA18 (secondary visual cortex), BA21 (medial temporal gyrus), and BA40 (intra-parietal sulcus). Frontal decrease and increase changes in right hemisphere corresponded to BA01 (primary somatosensory cortex), BA05 (superior parietal sulcus), BA10 (fronto-parietal cortex), BA06 (supplementary motor cortex), BA46 (medial prefrontal cortex), BA08 (pre-supplementary motor cortex), and BA09 (dorsolateral prefrontal cortex). Immediately within one minute of stopping the treatment sound (Figure 13), ongoing brain activity changes compared to baseline were present in the right temporal (T6), temporoparietal regions (Tp8) (BA47 ventero-lateral prefrontal cortex), BA21 (medial temporal gyrus), BA40 (intra-parietal sulcus), BA39 (angular gyrus), BA19 (cuneus)], frontal regions (Fz and AFz) [BA10 (fronto-parietal cortex), BA09 (dorsolateral prefrontal cortex), BA46 (medial prefrontal cortex), BA06 (supplementary motor area)], and in the left parietal regions (P3, P5, and Po3) [BA37(occipital-temporal cortex), BA39 (angular gyrus), BA19 (cuneus), BA05 (superior parietal sulcus)]. A right hemispheric dominance switch was observable once the treatment sound was stopped. There were long-term sustained changes observed in brain activity following the three-month feasibility trial (Figure 14). Resting-state (no sound) measurements of participants at 3 months compared to baseline showed greater decrease activity in the right parietal regions (P6, P8, and Po8) close to temporal sites; greater increase activity in right parietal regions was also observable close to central sites and centro-parietal regions, with a robust right hemisphere dominance.

Figure 10, Figure 11, Figure 12, Figure 13 and Figure 14 divided EEG channels into five sites for both hemispheres (left and right) with respect to their topological information, including: Yellow color: Left and right frontal (Fp1, AF3, F5, F3, F1 and Fp2, AF4, F6, F4, F2); green color: Left and right frontocentral (FC5, FC3, FC1, C5, C3, C1 and FC6, FC4, FC2, C6, C4, C2); purple color: Left and right temporal (F7, FT7, T7, TP7 and F8, FT8, T8, TP8); blue color: Left and right centroparietal (CP5, CP3, CP1, P7, P5, P3, P1 and CP6, CP4, CP2, P8, P6, P4, P2); pink colour: Left and right occipitoparietal (PO7, PO5, PO3, O1, and PO8, PO6, PO4, O2).

#### 3.3.6. Qualitative Analysis (Participant Interview Excerpts Attached as Appendix A)

Qualitative interviews were obtained from 17 out of the 18 participants. One participant chose not to take part in the interviews but completed all other outcome measures and was consistent in sound use during the feasibility trial.

A general theme was change in tinnitus—many participants reported in the interview that they felt their tinnitus did not change overall over the 3-month trial, while others felt their tinnitus was more subdued or calmer/less intrusive. Two participants felt the tinnitus became louder. There was no particular trend of sound intervention type on reported perceived benefit. 

Participants were also asked if they perceived their tinnitus characteristics had changed through the trial. Seven participants found their characteristics did not change. The remaining participants felt like tinnitus was less noticeable or lower in volume (6 participants), tinnitus was more intense (2 participants); interestingly 3 participants noticed that the tinnitus became more varied in pitch:

*“It seems that the lower sound is more frequent and the higher sound only occasionally. Reverts to higher and more annoying pitch when tired or continuing to process thoughts when going to sleep”*

*[Fan] [Participant #2]*

*“Less uniform sound, more variation in pitch and sounds like more than one sound”*

*[Birds] [Participant #4]*

*“Overall enjoyed the sound but did not change tinnitus much—only changed from multiple component sound to single electronic scream—but can’t tell if due to life stress”*

*[Birds] [Participant #6]*

Although participants reported their overall tinnitus did not change, 11 participants felt like the environmental sound interacted with their tinnitus and aided in providing benefit:

*“Yes, when I missed a few days, it got worse so I have been more careful to listen every day, since then it has decreased and evened out. Strong left-sided tinnitus had more distinct ringing has now stopped, seemed to have helped with that.”*

*[Water sound/Rain] [Participant #3]*

*“Yes, it is much calmer after a session. Hardly noticing the tinnitus, change in loudness from perceived 9/10 to 2/10. The location is still the same. Much lower and worked better.”*

*[Water sound/Rain] [Participant #9]*

*“I was thinking today how the tinnitus has become less noticeable during the day. Enjoyed water sound more than tinnitus. Like the idea of sound covering up tinnitus. Tinnitus is not unnoticeable, just acceptable—more relaxed and readier to try ongoing management.”*

*[Water and Bird] [Participant #10]*

Four participants did not feel like the environmental sound interacted with their tinnitus in any way, and two participants felt like the sound made their tinnitus worse/were more aware of tinnitus.

*“I am more aware of tinnitus as paying attention to it when filling in questionnaires.” [Cicadas] [Participant #8] “Will get a residual hum in head for 5 min after finishing.”*

*[Water sound/Rain] [Participant #15]*

The vast majority of participants reported that the environmental sound was pleasant to listen to and that they enjoyed listening to it (10 participants) or that the sounds were neutral and did not evoke any emotional affect (4 participants). Two participants reported variability in the sounds but did not relate this to any emotional affect (2 participants). One participant reported extended listening to the sound to become annoying. 

The home environments in which the sound therapy was administered during the three-month trial had a high level of consistency between participants and always involved quiet environments. The most commonly reported environments were: While reading, quiet household chores and cooking, on the computer doing office/desk work, or in bed at night.

When examining any major life circumstance changes/events that may have occurred during the trial period, the majority of participants reported no such changes. One participant reported a positive change in their life circumstances:

*“Yes, I have left my job of 23 years and am 2 days into a less demanding job, to a much less stressful environment and workload.”*

*[Water sound/Rain] [Participant #9]*

Four participants reported negative events/changes in circumstances:

*“Holiday stress that causes jaw clenching and teeth grinding, not being in my own bed causing sore stiff neck.”*

*[Water sound/Rain] [Participant #3]*

*“Yeah absolutely, I’ve gotten super stressed about university because tests and deadlines are coming up and I’m so behind. My sleep schedule is completely out of whack, I’m struggling to stay hydrated, I haven’t been exercising much, etc. I’ve been anxious and demotivated and depressed.”*

*[Cicadas] [Participant #5]*

*“My father died and I was helping arrange his memorial, which was stressful, involved international travel, an over-full house and upset routine. Finding an hour of quiet was at times impossible.”*

*[Birds] [Participant #6]*

*“For the first 6 weeks, sometimes the tinnitus wasn’t there at times. The silence was very prominent, marked. About 4 weeks ago, there was work tension and noticed an increase in sound. Also flew down to Wellington 2 weekends ago—in middle of tension period—not sure if flying might have had an effect.”*

*[Water sound/Rain] [Participant #14]*

## 4. Discussion

This study investigated the benefits and neurophysiological basis of passive perceptual training and informational counseling to recategorize phantom perception as a more real auditory object. This was hypothesized to reflect perceptual recategorization processes at the level of later stages of auditory processing, thereby relieving cognitive resources and the impact of tinnitus on quality of life. 

Overall, the behavioral, qualitative, and electrophysiological results from this study are encouraging. Behavioral results showed a significant reduction in tinnitus impact of life over the three months of trial. This particularly related to aspects of tinnitus intrusiveness and ability to concentrate with tinnitus. Under the recategorization hypothesis proposed, one interpretation for this might be that the brain is not needing to allocate greater attentional resources in order to try and categorize tinnitus into an auditory object, less prediction errors are generated once the brain starts to ‘think’ of the tinnitus as another environmental sound. This allows for freeing up of global cognitive resources and may result in better ability to concentrate as well as explain for the intrusiveness of the tinnitus signal, observed as significantly reduced tinnitus ‘strong’ magnitude ratings and greater ability to ‘ignore’ tinnitus in the rating scales for tinnitus severity. Impaired memory and cognitive processing of short-term tasks have been observed commonly among individuals who experience greater tinnitus distress. Using psychoacoustic tinnitus matches, it was observed that there was a significant reduction in the level of sound needed to mask tinnitus at 3 months compared to baseline, by approximately 6 dB SL; a reduction in tinnitus magnitude following auditory perceptual training. 

Under the UVH, the psychological phenomenon whereby perceptual stimuli that are at the ambiguous boundary on a continuum in “human” and “non-human” categories give rise to a strong, negative affective responses [32], it is possible for participants to experience an increase in negative emotions or a decrease in positive emotions for the first versions of sound which are more ambiguous compared to later versions which strongly resemble environmental sound (non-human categorization) An increase in negative emotions at 2 weeks and 1 month were observed for the ‘Water and Bird’ sound predominantly. There was a decrease in negative emotions at 2 months and 3 months, less than baseline. In addition, a similar effect was seen for changes in TFI concentration scores over time, there was an initial increase in impact of tinnitus on concentration for 2 weeks and 1 month for ‘Water and Bird’ sound, then a decrease at 2 months and 3 months so it fell lower than baseline. It is not possible from this study alone to discern whether this 1–2-month mark is indicative of changes in perceptual categorization from tinnitus to environmental sound, but it is interesting to note, as well the fact it was present predominantly for only one of the five environmental sounds administered in the trial.

Another finding is related to changes in the perceptual quality of tinnitus. Albeit being a minority of the participants, some individuals reported new tones to appear in their tinnitus or for it to become less uniform. In psychoacoustic matching, 15 participants reported a change in tinnitus pitch, and 3 participants reported a change in tinnitus location. Those who had right-sided tinnitus had significantly greater baseline stress compared to left-sided tinnitus at baseline and at 3 months. 

Interestingly, no other domains changed significantly, including emotional domain (this was marginally significant and showed reduced emotional impact at 3 months compared to baseline) and quality of life domains. This is interesting as, in the qualitative interviews, the majority of participants preferred having the environmental sound and reported the sounds helped them to relax and were very pleasant. This dual-mode of sound therapy action has been observed in past studies [65,66,67] and isolated as the ‘context of sound’ effect and ‘presence of sound’ effect pathways by which sound therapy may be beneficial [21]. Conversely, when emotional affect was measured using the PANAS scale, there was the inverse effect observed—positive emotions were less likely at 3 months compared to earlier time frames following sound therapy. This effect seems to be driven by the changes of one participant particularly, discussed more in detail in a later section. 

In terms of the effect of intervention sound, the effect of tinnitus on concentration seemed to show the greatest difference between sounds, i.e., different sounds seemed to interact differently with the ability of participants to concentrate on tasks or focus on work. 

Fan sound led to decreased positive emotion scores at the 1-month mark compared to other intervention sounds. None of the changes carried on to the 3-month mark, however, thus overall, the amount of positive emotions experienced did not change between intervention sound types during the trial. 

The behavioral changes observed in this study may also provide an interpretation for the acute and chronic/sustained brain activity changes observed using spatiotemporal spiking modeling architecture. Particularly, perceptual changes with auditory therapy may correlate with changes in activation of neural tinnitus networks relating to attention and discriminatory judgments (e.g., dorsal attention network, precentral gyrus, ventral anterior network). Steady-state functional networks of tinnitus [68] include the DMN (posterior cingulate, bilateral superior frontal gyrus, medial frontal gyrus, angular gyrus—most active at rest and reduced when attention or goal-directed behavior is present), limbic network, auditory network, visual network, attention network (especially dorsal attention (bilateral intra-parietal sulci, ventral precentral gyrus, middle frontal gyrus, frontal eye fields), and executive control of attention (middle, inferior, medial frontal gyrus and anterior insula) and visual network. 

During the acute stepped morphing, frontal decrease activity reflecting part of the DMN may reflect reduced activity as a result of the initial immediate presentation of external sound in the form of the tinnitus avatar. A greater left hemispheric predominance of temporo-parietal, parietal, and occipital activity was observed; the left hemisphere seemed to have more both decrease and increase changes while the right had only decrease changes observable. These regions are generally involved in spatial cognition, memory retrieval, attention and auditory and visual processing, recognition, and categorization. A few regions generally seem to relate to visual categorization and recognition, and the authors hypothesize whether this might relate to the brain trying to find accompanying visual information for the sound, which has now been introduced; while it resembles tinnitus, it is not identical to it completely and has an onset time. Left activity predominance is consistent with previous literature; regardless of tinnitus laterality and anatomical hemispheric differences, Schecklmann et al. (2013) observed an overactivation of the left Heschl’s gyrus compared to the right [69]. However, Geven et al. (2014) found in their PET study comparing tinnitus patients and control subjects without tinnitus that left primary auditory cortex activity was greater than the right regardless of whether tinnitus was experienced or not and may be related to the metabolic resting activity of the cortex (i.e., is a normal characteristic) [70]. 

As the sound morphed more towards becoming an environmental sound, there was greater brain activity change compared to the first version sound and which spread more across both hemispheres and shifted more towards parietal, centro-parietal, and parieto-occipital regions. Direct comparison of brain activity between the final stage morphing sound and initial stage morphing sound showed greater both decrease and increase changes in parietal, parieto-occipital regions of both hemispheres; while the regions involved in processing sound may be similar, there is greater bilateral hemispheric involvement as the sound morphs over time into natural environmental sound.

Immediately after stopping the stepped morphing sound, ongoing brain activity changes were observable in the right temporal; temporoparietal regions. This change in hemispheric activity may indicate residual inhibition effects of masking but also residual perceptual processing of the morphed sound by the brain. Greater resting-state decrease activity in the right parietal regions close to temporal sites was also sustained following 3 months of administration of the feasibility trial; there was greater increase in activity in right parietal regions close to central sites and centro-parietal regions. Tinnitus duration has been correlated positively with brain metabolic activity in the right hemisphere—corresponding to right inferior frontal, ventro-medial prefrontal, and posterior cingulate cortex regions (attentional networks) [69]. Given the average duration of tinnitus in participants was 20.5 years (albeit SD = 20.4), one interpretation is that the sound therapy, when administered over a longer period of time, recruited or interacted with this established right dominant network for ‘presence’ of phantom sound and in diverting attention away and thereby aided in reducing tinnitus outcome measures.

Hofmeier et al. (2018) have observed a right-hemisphere correlation between tinnitus loudness and auditory perceptual difficulty among tinnitus sufferers, independent of differences in hearing thresholds [71]. This correlation was linked to reduced and delayed sound-induced suprathreshold auditory brain responses (ABR wave V) and reduced BOLD activity in sound detection regions (posterior insula, hippocampus) and cortical auditory regions. A significant reduction in positive interhemispheric connections was also observed among tinnitus sufferers; it is possible our results may also partially reflect this and normalization of hemispheric involvement with the presence of treatment sound. 

The majority of participants felt environmental sound interacted with their tinnitus in a positive way, but many said they experienced no change in tinnitus severity. The characteristics of tinnitus were also reported to change, such as pitch, variation in tones, uniformity of sound that may not correlate with reported intensity changes measured in the quantitative outcome measures. The environmental sounds were judged by the majority of participants as being pleasant and something they wanted to listen to; this is encouraging for this feasibility study and in designing longer-term trials. A key question not answered by this research is whether the effects seen were due to recategorization or another mechanism, possibly simply masking or some emotional benefit from listening to a pleasant sound. There was no healthy control group thus, we cannot say unequivocally which effects are specific to tinnitus. This needs to be tested in a controlled trial, a divergence in EEG and psychoacoustic tasks is needed to elucidate whether the morphing signal has any benefit above Broadband Noise (BBN) and what the basis of any divergence may be due to. The passive training approach used in this study is feasible and provides positive results, but it is not known how well the method compares to conventional sound therapy. 

## 5. Conclusions

This is the first study that attempts to recategorize tinnitus using passive auditory training to a sound that morphs from resembling the person’s tinnitus to a natural sound. Acute and chronic passive training to a sound that is cross-faded between a tinnitus avatar and a natural sound showed reduced TFI and rating scores compared to a baseline measure. Qualitative analysis found that the environmental sound interacted with the tinnitus in a positive way, but participants did not experience a change in severity, however, characteristics of tinnitus, including pitch and uniformity of sound, were reported to change. The results indicate the feasibility of the method and preliminary evidence of changes in brain activity modeled from EEG data that suggest activation of neural tinnitus networks change and greater bilateral hemispheric involvement as the sound morphs over time into natural environmental sound; particularly neural changes relating to attention and discriminatory judgments (dorsal attention network, precentral gyrus, ventral anterior net-work).The use of recategorization perceptual training as a means to reduce tinnitus warrants further exploration to elucidate whether the approach used differs in effect and mechanism from conventional (BBN) sound therapy.

## Data Availability

Ethical approval for data sharing was not obtained, and thus the raw data are unavailable. Some software modules used for the implementation of the applied method can be found at http://www.kedri.aut.ac.nz/neucube accessed on 1 January 2018.

## Appendix A

Qualitative interviews: List of questions and patient-reported ratings of tinnitus are provided in Appendix A.

**Table:**

How is your tinnitus at 3 months? Tinnitus is like always [Cicadas] [#1] Less intrusive mostly [Fan] [#2] Variable but less intrusive than before [Water sound/Rain] [#3] Seems a little louder at times [Birds] [#4] Not very good, it’s louder [Cicadas] [#5] Noticeable and consistent [Birds] [#6] No change [Birds] [#7] Have not noticed any significant difference [Cicadas] [#8] Much calmer [Water sound/Rain] [#9] Mostly it seems somewhat subdued; I am aware of it, it just doesn’t seem so aggressive. Unless of course I get stressed. Then my head pounds, and the tinnitus is louder [Water and Bird] [#10] Less of a problem than usual [Birds] [#11] Constant. No change [Birds] [#12] No change. Still annoying! [Water sound/Rain] [#13] Much the same [Water sound/Rain] [#14] Just the same. Have several tinnitus components: hissing, bells, buzzing, chiming, tones [Water sound/Rain] [#15] About the same [Water sound/Rain] [#17] Much as usual. I may be less aware of it [Water sound/Rain] [#18]
How is the sound? (you can comment on any features such as quality, annoyance, pleasantness of the sound, etc.) Sound is getting annoying after 30 plus minutes, quality is OK I guess. Boring as ever [Cicadas] [#1] More subdued/lower/less intrusive [Fan] [#2] It is now balanced with a very high-pitched lining that is constant [Water sound/Rain] [#3] A few more tones in it—and sound varies a little more [Birds] [#4] I kind of got used to it and maybe it’s just my imagination but it seems to help me ignore the tinnitus a little better [Cicadas] [#5] Quality is very good, although I have trouble fitting the left earphone snugly—my ears are tricky. The sound is clear, although there is sometimes interference when the cables to the ear plugs rub against clothing or hair [Birds] [#6] Not too annoying but wouldn’t want it to be any louder. The sound wasn’t different enough from original bird noise and also sounded like the clip was created artificially by modulated, different bird noises merged. Seemed repetitive [Birds] [#7] OK [Cicadas] [#8] Very pleasant. Enjoyed the sound and feels there is no need to improve anything. [Water sound/Rain] [#9] I enjoy listening to the sounds, especially when they have all mingled together. It is very relaxing. I sometimes would like the sound to last longer. I used to listen to, “forestbythesea” frequently, now I find I listen a lot less. It is, however, my first go to tune when I am stressed, as it masks the tinnitus wonderfully. File was fine, possibly bit repetitive (too much water) but finds it builds to a crescendo so needed to make it lower in volume [Water and Bird] [#10] Easy to listen to. Does not interfere with other activities [Birds] [#11] Enjoy sound track. The sounds are pleasant and nice background noise, helped to relax. [Birds] [#12] I feel really content listening to the sound. It sounds similar to the running water in a pool I was staying by on my recent holiday so conjures up happy memories [Water sound/Rain] [#13] It’s pleasant enough, not a problem [Water sound/Rain] [#14] Fine [Water sound/Rain] [#15] It’s OK and quite nice to fall asleep with [Water sound/Rain] [#17] It is OK [Water sound/Rain] [#18]
Do you feel like your tinnitus (e.g., characteristics of your tinnitus such as the pitch, duration, fluctuation, intensity in different environments) has changed? Please describe. Has not changed [Cicadas] [#1] It seems that the lower sound is more frequent and the higher sound only occasionally. Reverts to higher and more annoying pitch when tired or continuing to process thoughts when going to sleep [Fan] [#2] Yes—left side is matching right, which is an improvement. Volume has decreased [Water sound/Rain] [#3] Yes—less uniform sound, more variation in pitch and sounds like more than one sound. Sound comes and goes a little more [Birds] [#4] It’s gotten worse, I feel like there are new tones appearing too, especially at night. If I start focusing on it then many new tones will appear that I didn’t notice before. I think it varies day by day slightly but on the whole, it got louder and harder to ignore, and I’m noticing it much more often [Cicadas] [#5] This trial it was difficult to judge if tinnitus changed because my father passed away 2 weeks in [Birds] [#6] No [Birds] [#7] Tinnitus has become more variable after beginning trial. At the moment it seems reasonably quiet. However, during trial I became more aware of it, as having to listen to it and thinking about whether it has changed when filling in questionnaires [Cicadas] [#8] My tinnitus is quieter since going on the 2nd version. Yes, less noticeable a lot of the time [Water sound/Rain] [#9] Perhaps the intensity of the tinnitus is slightly less. I haven’t noticed any change to the tinnitus in a stressful situation. I am acutely aware of the tinnitus, and know I need to calm myself asap [Water and Bird] [#10] I think the intensity is reduced [Birds] [#11] No change. Thought early on that tinnitus was louder after listening session but may be just greater awareness. Once I stopped “listening” to tinnitus seemed to go back to the usual [Birds] [#12] The volume in my right ear seems to have reduced in the last few weeks (probably settled down after long plane journey). The left ear is probably still louder than before I travelled but does have short periods of a lower (more usual) volume. I’m a lot more aware of it at night time. [Water sound/Rain] [#13] Occasionally it seems to be silent for a little while [Water sound/Rain] [#14] No [Water sound/Rain] [#15] Not really, it’s about the same loudness and more in my left ear as before [Water sound/Rain] [#17] No change. May be slightly less conscious of it [Water sound/Rain] [#18]
Do you feel like the sound has been interacting with your tinnitus since the last appointment? Please describe. Would say no [Cicadas] [#1] I think so. Tinnitus has only been noticeable perhaps when I am more stressed or tired?? [Fan] [#2] Yes, when I missed a few days it got worse so I have been more careful to listen every day, since then it has decreased and evened out. Strong left sided tinnitus had more distinct ringing has now stopped, seemed to have helped with that. [Water sound/Rain] [#3] I certainly here bird sounds outside a bit more—or at least I pay more attention to them [Birds] [#4] If I listen to the old [version] mp3, it really helps to ignore the tinnitus while it’s playing, and even if I try to notice my tinnitus ringing, it’s hard to actually hear it. The new one is ok too [Cicadas] [#5] No. Overall enjoyed the sound but did not change tinnitus much—only changed from multiple component sound to single electronic scream—but can’t tell if due to life stress [Birds] [#6] Yes—tinnitus is less noticeable while listening. The first set of sounds had a certain amount of effectiveness, the tinnitus was fading in to environmental sound. The later sounds I found tinnitus faded out only because of concentrating on the sound, not due to ‘auditory’ interaction with tinnitus [Birds] [#7] I am more aware of tinnitus as paying attention to it when filling in questionnaires [Cicadas] [#8] Yes, it is much calmer after a session. Hardly noticing the tinnitus, change in loudness from perceived 9/10 to 2/10. The location is still the same. Much lower and worked better. [Water sound/Rain] [#9] I was thinking today how the tinnitus has become less noticeable during the day. Enjoyed water sound more than tinnitus. Like the idea of sound covering up tinnitus. Tinnitus is not unnoticeable, just acceptable—more relaxed and ready to try ongoing management [Water and Bird] [#10] I feel that the MP3 sound is having a beneficial effect [Birds] [#11] No change. Tinnitus hasn’t changed, and even when birds chirping on sound file the tinnitus still sits in the background [Birds] [#12] I find that when I am getting frustrated with my tinnitus, I choose to go and listen to the sounds to distract myself from it. I sometimes wonder if the sounds are causing the ongoing increased volume but it may just be co-incidental with the travel. I do find that I seem to be going to sleep much faster than I normally would when listening to the sounds. When sound is playing, tinnitus is not as bothersome/audibility is reduced. When tinnitus is very loud, want to listen to it. [Water sound/Rain] [#13] They just seem to be alongside each other and it’s ok [Water sound/Rain] [#14] Will get a residual hum in head for 5 min after finishing [Water sound/Rain] [#15] I think there is a slight improvement, in the sense of a connection between the water sound and the tinnitus [Water sound/Rain] [#17] Maybe. Slightly less conscious of my tinnitus [Water sound/Rain] [#18]
What environments have you been using the sound in? Quiet environments, e.g., computer work, reading book/reading on the computer, quiet housework, cooking [Cicadas] [#1] Mostly reading. A couple of times when doing mindless household chores [Fan] [#2] In bed at night (before I go to sleep or as I go), when I go for a walk, or sitting at my desk [Water sound/Rain] [#3] Quiet office at work [Birds] [#4] At my desk while studying usually. Sometimes it’s hard to find a quiet hour because I live with my girlfriend and she always wants to talk to me about something [Cicadas] [#5] At home with no radio or television, sometimes reading or on a computer. Occasional conversation with a family member [Birds] [#6] Working at desk, reading [Birds] [#7] At home [Cicadas] [#8] Mainly at home alone cooking or reading or doing crossword [Water sound/Rain] [#9] Generally, I listen before going to sleep, then I replay the sounds as I go to sleep. I feel I have to listen to the sounds continuously to get the full benefit, not stop and start. Sometimes I have uninterrupted time to listen during the day. If I wake in the early morning, and the tinnitus is annoying, I will play the sounds to relax me [Water and Bird] [#10] Usually at home when reading. Occasionally while TV is on [Birds] [#11] Used them mainly in quiet environments, sometimes in early morning or in mid-late afternoon, when reading [Birds] [#12] I mostly listen to them at home during the day when going about normal activities—housework, admin, working from home on computer. A few times I have listened when out walking and I have also got into the habit of listening to them when I go to bed at night. Usually do other things while listening to it, walking children to/from school, house work, reading book (don’t really pay attention when reading). Prefer the birds/more environmental sounds—rain sound is more background [Water sound/Rain] [#13] Mostly when I am meditating [Water sound/Rain] [#14] In the lounge and mostly in the bedroom before I go to sleep [Water sound/Rain] [#15] Mainly at night [Water sound/Rain] [#17] At my desk when working on my computer [Water sound/Rain] [#18]
Are there any major changes in your life circumstances or incidents since the last appointment or email follow-up, which you believe may have had an influence on your tinnitus? Only change was school holidays as well as my ones [holidays] (3 weeks) [Cicadas] [#1] No [Fan] [#2] Holiday stress that causes jaw clenching and teeth grinding, not being in my own bed causing sore stiff neck [Water sound/Rain] [#3] No [Birds] [#4] Yeah absolutely, I’ve gotten super stressed about university because tests and deadlines are coming up and I’m so behind. My sleep schedule is completely out of whack, I’m struggling to stay hydrated, I haven’t been exercising much, etc. I’ve been anxious and demotivated and depressed [Cicadas] [#5] My father died and I was helping arrange his memorial, which was stressful, involved international travel, an over-full house and upset routine. Finding an hour of quiet was at times impossible [Birds] [#6] No [Birds] [#7] No [Cicadas] [#8] Yes, I have left my job of 23 years and am 2 days into a less demanding job, to a much less stressful environment and workload [Water sound/Rain] [#9] No [Water and Bird] [#10] I don’t believe so. I have started regular Gym activity during this trial [Birds] [#11] No incidents or circumstances [Birds] [#12] No [Water sound/Rain] [#13] For the first 6 weeks, sometimes the tinnitus wasn’t there at times. The silence was very prominent, marked. About 4 weeks ago, there was work tension and noticed an increase in sound. Also flew down to Wellington 2 weekends ago—in middle of tension period—not sure if flying might have had an effect [Water sound/Rain] [#14] No [Water sound/Rain] [#15] No [Water sound/Rain] [#17] No changes [Water sound/Rain] [#18]
How is the loudness of the sound? Would you like to adjust the sound level? The new sound is good, clear and partly loud [Cicadas] [#1] Fine. I vary the volume when I feel it needs adjusting [Fan] [#2] I adjust if I need to [Water sound/Rain] [#3] It’s fine [Birds] [#4] It’s fine I just set the volume [Cicadas] [#5] The volume varies throughout the track, and is sometimes quite soft and other times loud, but it is no more than attention grabbing when the volume changes [Birds] [#6] Very quiet in the first few minutes, later its OK [Birds] [#7] I am able to adjust the sound if I wish [Cicadas] [#8] It is fine [Water sound/Rain] [#9] I can adjust the sound level through my headphones [Water and Bird] [#10] I need to turn it up at first to hear it then it seems to get louder after a while [Birds] [#11] Can hear it OK [Birds] [#12] I’m happy with the loudness. I adjust the volume depending on what earpieces I am using but find it matches the loudness of my tinnitus well [Water sound/Rain] [#13] OK [Water sound/Rain] [#14] No thank you [Water sound/Rain] [#15] It’s OK, I can adjust to suit [Water sound/Rain] [#17] It is OK, environmental sound content seems greater in volume than tinnitus [Water sound/Rain] [#18]

Note: Participant #16 LS did not partake in the qualitative interviews in the trial but completed quantitative outcome measurements.
